# Barriers and Facilitators of Obtaining Social Determinants of Health of Patients With Cancer Through the Electronic Health Record Using Natural Language Processing Technology: Qualitative Feasibility Study With Stakeholder Interviews

**DOI:** 10.2196/43059

**Published:** 2022-12-27

**Authors:** Jordan Alpert, Hyehyun (Julia) Kim, Cara McDonnell, Yi Guo, Thomas J George, Jiang Bian, Yonghui Wu

**Affiliations:** 1 Cleveland Clinic Center for Value-Based Care Research Cleveland, OH United States; 2 College of Journalism and Communications University of Florida Gainesville, FL United States; 3 Health Outcomes and Biomedical Informatics, College of Medicine University of Florida Gainesville, FL United States; 4 Division of Hematology and Oncology, Department of Medicine College of Medicine University of Florida Gainesville, FL United States

**Keywords:** natural language processing, qualitative, social determinants of health, electronic health records, cancer, technology, education, patient, clinical, communication, data

## Abstract

**Background:**

Social determinants of health (SDoH), such as geographic neighborhoods, access to health care, education, and social structure, are important factors affecting people’s health and health outcomes. The SDoH of patients are scarcely documented in a discrete format in electronic health records (EHRs) but are often available in free-text clinical narratives such as physician notes. Innovative methods like natural language processing (NLP) are being developed to identify and extract SDoH from EHRs, but it is imperative that the input of key stakeholders is included as NLP systems are designed.

**Objective:**

This study aims to understand the feasibility, challenges, and benefits of developing an NLP system to uncover SDoH from clinical narratives by conducting interviews with key stakeholders: (1) oncologists, (2) data analysts, (3) citizen scientists, and (4) patient navigators.

**Methods:**

Individuals who frequently work with SDoH data were invited to participate in semistructured interviews. All interviews were recorded and subsequently transcribed. After coding transcripts and developing a codebook, the constant comparative method was used to generate themes.

**Results:**

A total of 16 participants were interviewed (5 data analysts, 4 patient navigators, 4 physicians, and 3 citizen scientists). Three main themes emerged, accompanied by subthemes. The first theme, importance and approaches to obtaining SDoH, describes how every participant (n=16, 100%) regarded SDoH as important. In particular, proximity to the hospital and income levels were frequently relied upon. Communication about SDoH typically occurs during the initial conversation with the oncologist, but more personal information is often acquired by patient navigators. The second theme, SDoH exists in numerous forms, exemplified how SDoH arises during informal communication and can be difficult to enter into the EHR. The final theme, incorporating SDoH into health services research, addresses how more informed SDoH can be collected. One strategy is to empower patients so they are aware about the importance of SDoH, as well as employing NLP techniques to make narrative data available in a discrete format, which can provide oncologists with actionable data summaries.

**Conclusions:**

Extracting SDoH from EHRs was considered valuable and necessary, but obstacles such as narrative data format can make the process difficult. NLP can be a potential solution, but as the technology is developed, it is important to consider how key stakeholders document SDoH, apply the NLP systems, and use the extracted SDoH in health outcome studies.

## Introduction

The World Health Organization defines social determinants of health (SDoH) as “*non-medical factors that influence health outcomes*, *such as where people are born*, *live*, *learn*, *work*, *worship*, *and age that affect health*, *quality-of-life*, *and risks*” [1]. They can broadly be categorized as (1) health care access and quality, (2) education access and quality, (3) social and community, (4) economic stability, and (5) neighborhood and built environment [2]. Within these 5 key areas, other factors, such as smoking status, substance use, homelessness, and alcohol use are the most frequently studied SDoH categories [3]. Health outcomes are impacted by SDoH in various ways. For example, data show that there is a strong correlation between socioeconomic status and diabetes [4], frequency of health care visits [5], and mental health [6]. In fact, clinical and medical care only accounts for 10%-20% of an individual’s modifiable determinants to healthy outcomes, while the other 80-90% are SDoH [7].

Although the characteristics about patients’ lifestyles and behaviors have been included in the medical record since the origin of documentation in the 1800s [8], the shift to electronic records held the promise of improving the integration of SDoH into health care delivery systems [9]. Effectively leveraging SDoH within the electronic record can yield many benefits, including improved diagnosis and treatment plan, resulting in better health outcomes [3]. However, despite the rapid expansion of SDoH documentation tools in electronic health record (EHR) systems, difficulties remain about how to effectively capture and utilize SDoH data [10,11]. For instance, a systematic review of social determinants research and data quality found that data from the EHR are often inaccurate, incomplete, and incompatible [12]. While SDoH data can be derived from structured fields, clinicians often do not use them [10], and instead enter SDoH-related data into their notes. One potential way of documenting SDoH in EHRs is to use the International Classification of Diseases, Tenth Revision, Clinical Modification Z codes (Z55-Z65), since they are intended to document patients’ SDoH related to their socioeconomic, occupational, and psychosocial circumstances [13]. We conducted a retrospective analysis of EHR data between 2015 to 2018 using a large collection of EHRs from the OneFlorida Clinical Research Consortium and found a low rate of usage for these Z codes (270.61 per 100,000 at the encounter level and 2.03% at the patient level) [13]. Clinicians often document SDoH in clinical notes; however, they were not collected in a systematic, structured format, posing further challenges and limits to their usage [3,5].

To better capture SDoH data, studies highlight natural language processing (NLP) as an effective tool for extracting insights from unstructured data [5]. NLP refers to a branch of artificial intelligence that enables computers to understand text in the same manner as humans [14]. NLP can extract SDoH data from narrative clinical notes into discrete variables [3], which can aid in the development of screening tools, risk prediction models, and clinical decision support systems. For example, including SDoH in risk prediction models not only improves model accuracy for hospitalization and death but also produces outcomes comparable to clinical factors [15]. Moreover, NLP produced a nearly 90-fold increase in identifying patients with significant SDoH problems compared to using structured EHR data elements alone [16]. We have systematically reviewed recent NLP studies for the extraction of SDoH [3] and developed NLP systems to identify SDoH from clinical notes [17]. Further, we have also assessed the extraction rates in patients with lung cancer [18] and studied how SDoH influence disparities of treatment selections in patients with diabetes [19].

Studies from our group and others show that it is feasible to use NLP to extract SDoH from clinical notes, but it is critical to understand what type of SDoH to collect and the best way to collect such data [20] when developing NLP solutions for SDoH extraction. Further, SDoH data generated from NLP are more useful if key stakeholders could incorporate the data to study real-world health outcomes. For example, the NLP extracted SDoH needs to be normalized to clinically meaningful categories (eg, stable housing, shelter, and homeless) related to outcomes of interest for analysis. How to standardize and populate NLP extracted SDoH concepts to common data models defined by large clinical research networks such as the Patient-Centered Clinical Research Network and the Observational Medical Outcomes Partnership remains unsolved. Engaging diverse stakeholders who document and frequently use SDoH can produce many benefits. Currently, there is a dearth of literature examining stakeholders’ perceptions of SDoH generated from NLP. This gap has resulted in NLP technology being developed without considering best practices for the oncologists and analysts who are the users of these NLP systems. Thus, the objective of this study is to understand the feasibility, challenges, and benefits of developing an NLP system to uncover SDoH data in EHRs. To best understand the facilitators and barriers, qualitative interviews were conducted with four key stakeholders: (1) oncologists, (2) data analysts, (3) citizen scientists, and (4) patient navigators. The following research questions were explored: what factors facilitate obtaining SDoH data? What are the challenges to obtaining SDoH data? How can SDoH data from EHR be applied to health services research and clinical care?

## Methods

### Setting and Study Design

This study took place at the University of Florida Health (UFHealth) in coordination with the University of Florida Health Cancer Center in Gainesville, Florida.

### Participants

A form of purposive sampling, critical case sampling, was used to identify participants as the goal of the study was to assess a phenomenon of interest at its very early stages [21]. The 4 groups identified as critical for understanding how SDoH data can be effectively used were (1) oncologists, (2) data analysts, (3) citizen scientists, who are members of the community that engages with researchers to improve the quality of health care, and (4) patient navigators, who are typically nurses who help guide patients throughout the diagnosis and treatment processes. Inclusion criteria were being at least 18 years old, fluent in English, and willing to provide informed consent. Members of the research team identified individuals within UFHealth who had experience working with SDoH data on the basis of their job titles and referrals from the research team’s network. Once a master list was formed, they were contacted via email to participate. All participation was voluntary and was done without compensation.

### Data Analysis

An interview guide was developed by the research team using a grounded theory approach, in which interview questions were general to cover a wide range of experiences and also narrow to explore specific experiences [22]. Group discussions centered on existing literature formed the basis for initial questions. Modifications were made to tailor a subset of specific questions for each group. Sample questions can be found in Textbox 1. Semistructured interviews were conducted because they allow for detailed information about a phenomenon to be obtained [23], as well as for the ability to immediately ask follow-up questions for clarification [23]. All interviews were conducted by 2 of the coauthors (JA and HK) using videoconferencing technology. Upon verbatim transcription of the video recording, the lead author (JA) performed primary cycle coding by reading 2 transcripts from each group [24]. Once an initial codebook was generated using the qualitative software ATLAS.ti, another coauthor (HK) read the same transcripts as the lead author, as well as one additional transcript from each group. A list of preliminary themes was presented to all of the other coauthors, and modifications were made after discussions. The remaining transcripts were read and coded and subjected to a process of constant comparison [25], and themes were generated. Interviews and data analysis continued until thematic salience occurred through the criteria of repetition, recurrence, and forcefulness [26]. During the analysis stage, interviews continued until saturation of the data was achieved [27]. Finally, another coauthor (CM) reviewed several transcripts and coded each one as a validation strategy. To confirm the findings from our analyses, self-reflecting memos that were recorded during interviews were verified [28] and to ensure trustworthiness, in vivo quotes were included [29].

**Sample interview questions.**

**Oncologists**

- Describe the types of information you regularly gather during a typical interaction with a patient.
- How could we better obtain social determinant information from patients?
- What tool within the electronic health record (EHR) system would be helpful to improve the entry of social determinant variables?

**Citizen scientists**

- How do you think social determinants of health information is important for your health care provider?
- How does your provider usually ask about social determinants of health?
- What suggestions do you have for providers to more effectively learn about your social determinants of health?

**Data analysts**

- How can social determinants of health be important for health outcomes research?
- How do you work with other researchers and oncologists using data about social determinants of health?
- What role does the EHR system play in being equipped to provide data?

**Patient navigators**

- Describe your typical interaction with a patient.
- In your experience, are patients comfortable sharing information about social determinants?
- What is your experience reviewing a patient’s EHR with them?

### Ethical Considerations

This study was approved by the University of Florida institutional review board (IRB202002156). All procedures were performed in accordance with institutional guidelines regulations and human subject protections. Informed consent was reviewed with all participants prior to interviews and it was explained that participation was voluntary and they were free to withdraw at any time.

All data were transcribed and any identifiable information was removed from the record. Data were saved on secure Health Insurance Portability and Accountability Act–approved servers, and only members of the research team had access.

## Results

### Participant Characteristics

From August to September 2021, a total of 16 participants agreed to be interviewed (66% recruitment rate), consisting of 5 data analysts, 4 patient navigators, 4 oncologists, and 3 citizen scientists. All participants were based in the United States; their average age was 48 years, and most of them were female (63%) and White (69%). Among oncologists, the average time from fellowship was 24 years, ranging from 3 to 35 years. Interviews averaged 26 minutes in length and 160 pages of transcribed data were generated.

### Theme 1: Importance and Approaches to Obtaining SDoH

#### Overview

The main theme of the importance and approaches to obtaining SDoH emerged, summarized by 2 subthemes. The first subtheme, importance of SDoH, focuses on how doctors, navigators, and citizen scientists value SDoH data and why they think they are essential to be collected. The second subtheme, SDoH solicitation during patient-provider communication, addresses how SDoH are woven into discussions that occur between patients and other members of the health care team such as navigators, social workers, and nurses.

#### Importance of SDoH Data

Every participant (n=16) across all stakeholder groups agreed that SDoH data were very important for patient care. When asked to name a specific type of SDoH data that may not be particularly important, participants struggled to provide an example. Doctors and patient navigators expressed how SDoH played a major role in the delivery of care. For instance, one particular SDoH, proximity to the hospital, was deemed crucial because as doctor #9 said, “If the patient lives further away…they're less likely to make it to the appointments. Less likely to make it on time. More likely to miss appointments and have subsequent negative outcomes.” Another SDoH, social structure, was related to helping patients get to the hospital as well as managing side effects. Doctor #9 said, “Our treatments can be physically and mentally debilitating…they see me or my colleagues for maybe 15 to 20 minutes…They spend the majority of time outside of our clinic and having someone that they can rely on to help them with their symptoms.” Citizen scientist #4 recalled her experience as a patient and the importance of social support. She said, “Thank God I had the support of my family, my mom, my sister. That was a big one for me just with living with [the disease].” Doctors can use information about patients’ social structure to effectively communicate with patients and family members, as well as bring clarity as to who can assist patients the most. For example, doctor #10 provided the following example:

The individual that's accompanying the patient every day to clinic, is probably not the one who's doing a lot of the heavy lifting or at least a lot of the heavy organizing. Maybe he is there for the day-to-day things but he's not he's not the person that needs to be involved with major life transition points in her care. So that's been, that's been really, really insightful.

Other SDoH considered important were income levels and geographic location. All data analysts (n=5, 100%) mentioned that zip codes were the most frequently requested data point by health service researchers. Zip codes were powerful because they usually are connected to geography. Analyst #12 said, “Zip code is probably the most important one because you can link that back to job opportunities [and] someone's almost entire socio-economic status just from where they live.” Another analyst (#13) referred to how income and geography were connected due to “air quality [and] the stresses of the environment.”

#### SDoH Solicitation During Patient-Oncologist Communication

The process of soliciting SDoH information usually occurred during initial consultations. Traditionally, in a formal procedure conducted at the first visit, doctors ask standardized questions, prompted by smart forms in the EHR. Smart forms allow oncologists to enter data, usually in the form of a drop-down menu. However, time restraints are an obstacle during initial visits, resulting in not all SDoH being collected. Doctor #9 said, “It can be hard to get into everything in that first visit…that first visit is a pretty packed visit.” Doctors found that additional information about patients surfaced once the patient-oncologist relationship was better established. For instance, doctor #10 said, “You have to be prepared to listen for it (SDoH) and then and then take that opportunity since they brought it up to let your foot in the door and pursue it a little bit more.” Citizen scientists agreed with this sentiment and recognized the importance of doctors getting to know their patients. Citizen scientist #4 recommended that doctors should “make the patient feel like you're there for them. You want to know what's really going on, make them feel comfortable.” Doctor #9 said the following:

Subsequent visits I'm able to have more of that kind of conversational approach about asking them about other aspects of their care and I think that is also because…with subsequent visits, I’ve built that relationship. Sometimes it can be difficult to ask serious and personal questions to the patient with that first visit.

Patient navigators also obtain SDoH data from patients during conversations, but the nature of the navigator-patient relationship sometimes allows for the acquisition of more personal detail. Navigators often form a close bond with patients, which differs from patient-oncologist interactions. Navigator #8 mentioned that patients confide in them because of the high level of trust that is formed. Citizen scientist #5 reiterated this notion, saying, “Can you imagine your doctor asking ‘do you have enough money to buy groceries? Is your neighborhood dangerous? Is your sex life interesting?’ They don't go there.” Navigators either alert oncologists or enter SDoH data directly into the EHR under their own notes. Doctor #10 noted the importance of social workers discussing SDoH with patients because, in addition to helping with things like travel or insurance, they can also be “a shoulder for [patients] to cry on.” Overall, SDoH solicitation more realistically occurs on a continuous basis during the relationship between the patient and oncologist.

### Theme 2: SDoH Exists in Numerous Forms

#### Overview

While it is common to consider SDoH as medical information obtained during a consultation, it can transpire in other ways. The first subtheme, informal communication, describes how not all SDoH are included in the EHR, while the second subtheme, the need for SDoH expertise and knowledge, explains the ways in which SDoH can be uncovered.

#### Informal Communication

Although smart forms were cited as a sufficient method of capturing SDoH data, patient information can be communicated informally, placing the onus on oncologists to separately enter data that they deem significant. Doctor #9 commented, “If I do elicit something from [a] patient encounter that I think is really important, I'll put it in the note. But I think the issue is that there's no section in our typical note template.” As a result, doctors were uncertain about where to include such information in the note. Similarly, another doctor (#15) said, “I don't put it in the template…I put what I think is important to me [in] the first paragraph.” In addition to communication that occurred during examinations, SDoH can also be uncovered during alternative methods of communication. Doctor #10 said, “MyChart messaging [gave] me insights that I didn't already have in the clinic.” For instance, the doctor mentioned the following:

When there's consistently another caregiver in their social system that's engaging with us on the patient's behalf…maybe these are individuals that I haven't met…but clearly are intimately involved in the day-to-day support of the patient.

Since SDoH can come from many different sources, like messaging as well as additional notes input by navigators, it is imperative that they are properly included in the EHR. Although smart forms seem easier to enter SDoH data, doctor #10 noticed that “every discrete variable requires at least 15 clicks.” As a less time-consuming alternative, doctors will type in data, which are considered unstructured data, because the information is not necessarily included in smart forms. To get such data into the EHR, manual documentation is often required. It is common for oncologists to copy and paste previous notes, but doing so many omit new SDoH that arise during recent examinations.

#### Expertise and Knowledge Needed

Inputting data manually creates obstacles to extracting SDoH data. Data analyst #13 said, “Having unstructured data would be a lot more difficult for the end-user because a lot of researchers are not going to have skills…to try to actually get the data out that they need.” Deciphering unstructured SDoH data are problematic because as data analyst #12 asked, “A lot of that stuff (SDoH) is…stored in a notes section…how do we get it out of the notes and then how do we put it into a structured format?” Analysts are forced to get creative to identify SDoH that are often requested by researchers. For instance, data analyst #12 continued, “We don't have education status. We don't have income levels or anything for individual patients…we can infer that stuff from zip code but that only goes so far.” Doctor #10 stated, “We wouldn't even consider not documenting a patient's past surgical history, but [SDoH] are not captured in the [EHR] optimally…because it requires too much manual labor to type into and put into the system.”

When NLP was proposed as a solution, analysts were intrigued, but none of the 5 analysts had the expertise to extract data that way. Data analyst #17 said, “I know what NLP does and I have played with some stuff before, but it's not something that I do in my work.” Another analyst (#14) had no experience with NLP but said, “It's an interesting area of the field, but I haven't personally worked with it.” Doctors, like participant #15, were skeptical that NLP could be a solution soon. He said, “It's not ready for prime time, but there might be beta stuff that I'm just not aware of that's ready to go.”

### Theme 3: Incorporating SDoH Into Health Services Research

#### Overview

Given the challenges of obtaining and extracting SDoH, combined with the importance of SDoH to treat patients, it is necessary to discover methods of incorporating data into research to expand their impact. Two subthemes emerged that addressed possible solutions to improve how SDoH data can be incorporated into health services research and patient care: (1) empower patients and (2) actionable data.

#### Empower Patients

Truly understanding the patient as a person requires that SDoH, such as the neighborhood in which they reside, occupation, access to health, and social support, are identified and integrated into the care plan. Although oncologists solicit information from patients, there are opportunities to increase patient participation in the process. For instance, doctor #9 said, “We're always interested in getting information from the patients directly, but I think it's not a bad idea to have the patients voluntarily answer questions.” The doctor elaborated that surveys could be distributed via email up to a week before meeting with the doctor to understand more about the patient’s background and environment. Doing so would “take a lot of the burden off of [oncologists].” Another doctor (#15) thought that patients should be more involved and have the ability to clarify information about themselves because “there can be innocent errors or there's a certain amount of incorrect information all the time.” However, patients have other opportunities to fill out forms with information about themselves, but because they are optional, the majority of patients choose not to. As a result, a data analyst (#16) said, “We don't see much structured data… I do know there is this one survey that has many more structured questions, but not many people have filled [it] out.” Doctor #10 reinforced this notion by saying the following:

We've done away with a paper system of patients filling out information when they're in the waiting room which is another great missed opportunity for patients to get data into the system themselves that we could then verify.

#### Actionable Data

To involve patients, they must be made aware of how SDoH can affect them, but they also must understand the data. Citizen scientist #3 reflected upon her own family members who have high school educations and recognized that they might not have the ability to process health information as easily as others. Inputting or viewing SDoH data is even more helpful when patients can take action. For example, doctor #9 thought it would be beneficial for patients to have someone that they could talk with about SDoH for the following reason:

I think a lot of patients aren't aware of all the resources that are available to them whether it's patient assistance programs for drugs, or whether it's support groups… [or] financial programs.

NLP innovations were welcomed as a potential solution to aggregate individual-level SDoH data and as a way to make them more prominent for both patients and oncologists. Data analysts were enthusiastic about the prospect of acquiring additional data to work with, but were curious about the process of accurately validating data. Oncologists also realized the potential efficiencies of NLP. Referring to NLP, doctor #9 commented, “It's automatic. Doesn't require anything from us. I think it would be great…You're better able to incorporate the right management and the individualized care that the patient needs with regards to those social determinants.” Oncologists also considered where and how the data would be presented. For instance, doctor #10 speculated, “If the NLP system can identify the data can pull it out, what happens to it? Does it end up getting put into the discrete places in the EHR where it should have been to begin with?” Citizen scientists were cautiously optimistic about interpreting data derived from NLP. A citizen scientist (#5) provided the following example:

If I were looking at doctor's notes, and I said something about… [feeling] paranoid about my next-door neighbor…the word paranoid might appear in my record. What a misstatement that would have been for me…that could be coded into something that was not really a diagnosis for me. I worry about that process a whole lot.

## Discussion

### Principal Results

We interviewed 16 stakeholders with various involvements related to SDoH, including oncologists, data analysts, patient navigators, and citizen scientists. The findings revealed that no SDoH data point was deemed as unimportant. Geography, social structure, and income were most accessible and, therefore, used most often. Although the importance of SDoH on population health are known, SDoH have recently taken on a heightened level of importance, brought about by the COVID-19 pandemic. For instance, those with a lower socioeconomic status or those living with comorbidities were more vulnerable to infection [30]. Machine learning is frequently being used to uncover such SDoH [31], but it is not clear whether it can lead to improved patient experiences and more informed research [32]. Moreover, it is possible that techniques such as NLP may increase social biases [32]. Our study included the insights from multiple perspectives to help create a path for using NLP to take bias into account and can positively impact clinical encounters as well as health services research. Textbox 2 pairs each theme identified from the interviews with a direct actionable recommendation.

**Recommendations based on each theme.**

**Importance of social determinants of health (SDoH) data**

- SDoH should be prioritized and as much detail as possible should be included when inputting data into the electronic health record (EHR). Smart forms should be filled out and additional information about patients, such as their lifestyle and issues they are currently confronting, should also be added.

**SDoH solicitation during patient-provider communication**

- Although most SDoH are input into the EHR after the initial consultation, valuable SDoH are provided by patients in subsequent visits as the patient-provider relationship grows. Richer detail about patients’ lives should be continuously added to the EHR.

**Informal communication**

- While smart forms might guide the discussion, patients offer clues about their lifestyle throughout the visit. If a specific variable is not available via the smart form, it should be entered within the notes.

**The need for SDoH expertise and knowledge**

- Everyone involved in the research analysis process should be briefed and educated about technology to identify and extract SDoH.

**Empower patients**

- Increase patients’ awareness about how sharing SDoH to providers can positively influence their care.

**Actionable data**

- Involve patients in the implications of SDoH and how they can directly affect decisions that are made about their care.

Our results indicate that the method of obtaining SDoH from patients varied, as data arose during both formal and informal patient-provider communication and across the time line of care delivery. Physicians acquire SDoH during initial consultations, but due to time restraints, capturing all relevant SDoH is unlikely. SDoH can also be found within secure messages as well as during interactions with patient navigators. Previous studies show that patients’ self-disclosure depends on the gender of physicians and their own willingness to be open and disclose information to patients [33]. Additionally, the way questions are framed can dictate patients’ willingness to respond to sensitive questions about SDoH [34]. Therefore, relationship-centered communication has been suggested as a method for acquiring SDoH and sensitive information from patients [34]. This can occur by monitoring negative attitudes, displaying empathy, and honoring patients’ preferences [35]. During our interviews, patient navigators mentioned that they often receive patient information that is not disclosed to the doctor because of their close relationship with the patient.

Since most navigators have access to the EHR and can enter their own notes, it is important to use unstructured data to capture SDoH. NLP has produced valid results [36], but as algorithms become more accurate, it is necessary to involve data analysts who have historically worked with SDoH to support health services research. NLP requires a different skill set than what most data analysts are accustomed to. However, we discovered that data analysts were excited about the prospect of using NLP and enthusiastic about its capabilities. NLP should not be confined to programmers and machine learning experts. Data analysts should understand the capabilities and limitations of working with NLP while the technology is being perfected.

Lastly, interviews revealed that to better incorporate SDoH data into health services research and clinical care, patients should be empowered to understand how such data can impact their health and that valuable resources might be available upon disclosure of the information. Since time restraints are a common barrier to collecting SDoH [37], innovations that allow for patients to enter information about themselves directly into the EHR should be explored, allowing it to be entered once and be available to all members of their health care team. Patients often find errors in their health record [38], so the ability to review and edit or update the information is critical. While patient portal enrollment continues to increase, groups most affected by SDoH are often less likely to enroll in portals and have access to their health record [39].

### Limitations

This study includes several limitations. First, a fairly small sample size was used. Second, all interviews were conducted at the same health system. Therefore, observations from participants may be confined to the specific procedures of the health system and not applicable to stakeholders in other facilities. Third, there was an element of selection bias, as all participants volunteered to participate after being informed about the topic. It is possible that stakeholders who did not see value in NLP or SDoH, in general, chose not to participate.

### Conclusions

SDoH data are extremely valuable for patient care but can be difficult to access as a result of the way unstructured data are entered into the EHR. NLP can ease the burden on oncologists by identifying hidden SDoH data within the EHR while enabling analysts to easily extract requested data for health outcomes research. However, maintaining high levels of quality for SDoH data entered into the EHR is imperative. Processes should be developed to facilitate acquiring SDoH from patients as well as educating patients about the importance of SDoH.

## Abbreviations

EHR: electronic health record
NLP: natural language processing
SDoH: social determinants of health

## Data Availability

Deidentified data can be made available upon reasonable request.
